# Immune modulation of HLA-G dimer in maternal-fetal interface

**DOI:** 10.1002/eji.200737515

**Published:** 2007-07

**Authors:** Kimiko Kuroki, Katsumi Maenaka

**Affiliations:** Division of Structural Biology, Medical Institute of Bioregulation, Kyushu UniversityJapan

**Keywords:** HLA-G, HLA-G dimer, LILR, Immune suppression, Placenta

## Abstract

HLA-G is a non-classical human MHC class I molecule, which has several characteristics distinct from classical MHC, such as low polymorphism and restricted tissue distribution. HLA-G is expressed on placenta, thymus and some tumors. At the maternal-fetal interface, trophoblasts do not express major classical MHC class I molecules (MHCI), HLA-A and -B, to prevent normal T cell responses. Instead, HLA-G is expressed and can suppress a wide range of immune responses by binding to inhibitory immune cell surface receptors, such as leukocyte Ig-like receptor (LILR) B1 and LILRB2. HLA-G exists in various forms, including β2m-associated or -free disulfide-linked dimers that can be expressed either at the cell surface or in soluble form. However, until recently the physiological role of these different molecular forms has been unclear. In this issue of the *European Journal of Immunology*, one article demonstrates that the disulfide-linked homodimer of β2m-associated HLA-G is the major fraction expressed by trophoblast cells. The HLA-G dimer modulates the function of LILRB1-expressing antigen-presenting cells by principally binding to LILRB1. On the other hand, another recent report showed that β2m-free disulfide-linked HLA-G dimers are produced by villous cytotrophoblast cells. Taken together, these results provide strong evidence in support of the hypothesis that HLA-G dimers play a role in immune suppression at the maternal-fetal interface. Further in-depth investigation will help to clarify the precise mechanism of HLA-G receptor recognition and signaling *in vivo* and the role of these interactions in successful reproduction.

See accompanying article: http://dx.doi.org/10.1002/eji.200737089

During pregnancy, the semi-allogeneic fetus needs to avoid maternal immune responses. A specific immune suppression system employing the non-classical MHC class I molecule (MHCI), HLA-G 1, 2 has been adapted for this purpose. The gene encoding HLA-G is located within the MHC region; however, HLA-G has very low polymorphism by contrast with the high polymorphism of classical MHCI. Although HLA-G is expressed by placental trophoblast cells, these cells do not express major classical MHCI, HLA-A or -B, resulting in the inability to induce immune responses. Cell surface-expressed trophoblast HLA-G can suppress immune responses by binding to inhibitory receptors. The HLA-G receptors reported to date are the leukocyte Ig-like receptors (LILR) B1 and B2, killer cell Ig-like receptor (KIR) 2DL4, CD160 and CD8 2–4 (Fig. 1). Previous reports clearly demonstrate that LILRB1 and LILRB2 preferentially bind to HLA-G compared with other classical MHCI 5. Intriguingly, HLA-G is expressed in several unusual forms in addition to the conventional heterotrimer, associated with β2m and peptide, made by all classical MHCI. These include: (i) a disulfide-linked β2m-associated dimer; (ii) a β2m-free heavy chain, (iii) its disulfide-linked dimer, and (iv) domain-deleted and soluble splice variants 6. Previous *in vitro* studies using either recombinant proteins, transfectants or cell lines demonstrated that HLA-G can exist as a disulfide-linked dimer of the conventional β2m-associated form (and in some cases, trimeric and oligomeric forms) 7, 8. In this issue, Apps *et al*. 9 demonstrate that significant amounts of the β2m-associated HLA-G dimer are expressed at the cell surface of normal first trimester trophoblast cells. Furthermore, they also found that LILRB1 is the principal ligand for the HLA-G dimer. These results are consistent with previous studies showing that the HLA-G dimers bind with greater avidity to LILRB1/2 and signal more strongly through LILR than monomeric HLA-G and other classical MHCI 10, 11 (Fig. 1). Therefore, the HLA-G dimer presumably has a significantly pivotal *in vivo* role in maternal-fetal interface, in comparison with the monomeric form. In addition to the crystal structure of the HLA-G monomer 12, the recent structural study 10 showed that the disulfide-linked dimer exhibits an oblique configuration exposing two upward facing LILR/CD8 binding sites, readily accessible to receptors, explaining the increased avidity. On the other hand, the peptide-binding grooves are very close, possibly preventing T cell receptor binding (Fig. 1).

**Figure 1:**
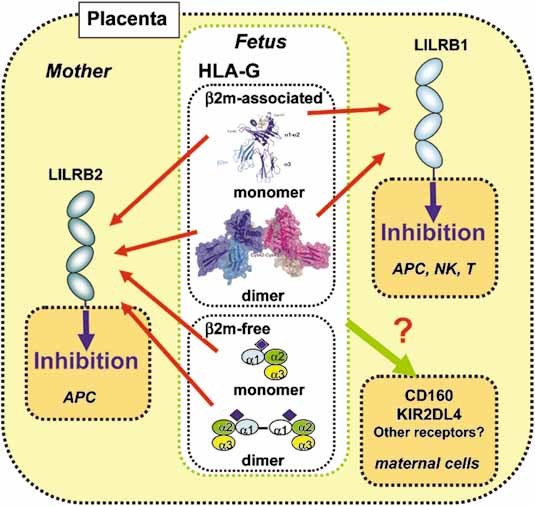
Receptor recognition of the different forms of HLA-G at the maternal-fetal interface. HLA-G can be expressed in various forms in placenta. The structures of β2m-associated HLA-G monomer and dimer are shown. LILRB1 (light blue), which is expressed on decidual antigen-presenting cells (APC) and subsets of NK and T cells, binds to only β2m-associated forms of HLA-G monomer and dimer. LILRB2 (dark blue), whose expression is restricted to decidual APC, binds to both β2m-associated and -free forms of HLA-G monomer and dimer. LILRB1 and LILRB2 presumably bind to HLA-G dimer more strongly than its monomer. The thickness of the arrows corresponds to the signal strength. Both membrane-bound and soluble forms likely inhibit the immune responses of LILRB-expressing cells. On the other hand, the HLA-G recognition of CD160 and KIR2DL4 still remains unclear.

There are some reports demonstrating the existence of a β2m-free heavy chain form of HLA-G. Gonen-Gross *et al*. 11 demonstrated placental expression of the β2m-free form of HLA-G by *in situ* immunostaining. Furthermore, Morales *et al*. 13 recently showed that villous cytotrophoblast cells (vCTB) express the dimeric form of β2m-free HLA-G. Based on these results, Hunt *et al*. 14 summarized the expression pattern of HLA-G variants in placenta. These results are different from those of Apps *et al*. 9 with regards to the β2m association of HLA-G dimer in placenta, and might be a consequence of the different cell populations used in each study. Recent reports 11, 15 have shown that LILRB2 can recognize the β2m-free MHCI, but LILRB1 cannot (Fig. 1). Therefore, β2m-free HLA-G confers an inhibitory effect selectively on LILRB2-expressing cells, such as antigen-presenting cells (APC). Furthermore, Kollnberger *et al*. 16 showed that β2m-free HLA-B27 dimer binds to KIR3DL2 and KIR3DL1 as well as LILRB2. Thus, it will be of great interest to see whether β2m-free HLA-G dimer binds to LILRB and KIR with greater avidity than monomer.

Emerging studies have revealed the potential roles of various forms of MHCI, including β2m-free MHCI (and its homodimer) and associated forms with other unrelated molecules 17. As described above, HLA-G can exist in similar forms, but possesses unique cysteine residues distinct from other MHCI, including Cys42, which is involved in disulfide-linked dimer formation without any conformational changes. Therefore, both β2m-associated and -free forms of the HLA-G dimer could have distinct roles in immune suppression from those of normal MHCI.

The soluble form of HLA-G (sHLA-G) is secreted and/or shed from the cell surface toward the decidua area. sHLA-G can inhibit immune responses of LILRB1/2-expressing cells in the decidua and can also interact with CD160 expressed on the surface of endothelial cells 18, possibly resulting in the anti-angiogenic effects on uterine vascular remodeling in pregnancy. By contrast, sHLA-G binds to KIR2DL4 at the cell surface and is internalized into endosomes, whereas the membrane-bound form of HLA-G cannot, thus stimulating pro-inflammatory and pro-angiogenic cytokines production, which would be helpful in increasing blood supply to the placenta 19. Using a killing assay, Yan *et al*. 20 revealed that the KIR2DL4 binding site is located in the C-terminal peptide-binding site of the HLA-G α1 helix. This suggests that the HLA-G dimer may not be able to bind to KIR2DL4 because of the close proximity of the peptide-binding grooves. However, without further biochemical evidence it is not possible to draw any definitive conclusions. Further in-depth investigations, especially comprehensive binding studies with recombinant proteins, will clarify the precise role of HLA-G monomer and dimer interactions with KIR2DL4 and CD160 in immune regulation.

Taken together, the current evidence and the results from Apps *et al.* 9 strongly suggest that LILR recognition of HLA-G dimers has a pivotal role to play in immune suppression at the maternal-fetal interface, possibly contributing to the prevention of pregnancy complications such as pre-eclampsia and recurrent abortion. Further detailed analysis of the interaction of HLA-G with LILR and KIR families, CD160 and maybe as yet-unidentified receptors will provide a clear molecular basis of how these interactions may be involved in immune suppression and the control of angiogenesis *in vivo*. These studies may also provide future avenues of research with potential clinical applications.

## Abbreviations

KIR: killer cell Ig-like receptor
LILR: leukocyte Ig-like receptor
